# Wind plants can impact long-term local atmospheric conditions

**DOI:** 10.1038/s41598-021-02089-2

**Published:** 2021-11-25

**Authors:** Nicola Bodini, Julie K. Lundquist, Patrick Moriarty

**Affiliations:** 1 National Renewable Energy Laboratory, Golden, 80401 CO USA; 2 Department of Atmospheric and Oceanic Sciences, University of Colorado Boulder, Boulder, CO 80309 USA; 3 Renewable and Sustainable Energy Institute, Boulder, CO USA

**Keywords:** Renewable energy, Atmospheric science

## Abstract

Long-term weather and climate observatories can be affected by the changing environments in their vicinity, such as the growth of urban areas or changing vegetation. Wind plants can also impact local atmospheric conditions through their wakes, characterized by reduced wind speed and increased turbulence. We explore the extent to which the wind plants near an atmospheric measurement site in the central United States have affected their long-term measurements. Both direct observations and mesoscale numerical weather prediction simulations demonstrate how the wind plants induce a wind deficit aloft, especially in stable conditions, and a wind speed acceleration near the surface, which extend $\sim 30$ km downwind of the wind plant. Turbulence kinetic energy is significantly enhanced within the wind plant wake in stable conditions, with near-surface observations seeing an increase of more than 30% a few kilometers downwind of the plants.

## Introduction

As wind flows past the rotating blades of a wind turbine, some of its momentum is devoted to moving the blades and generating electricity. As a result, the downwind flow is slower and more turbulent^1,2^. Assessing the characteristics of this wake has been identified as one of the grand challenges that wind energy science needs to face to drive innovation in the sector and meet future energy demand^3^. Wakes are particularly complex: their characteristics depend on the incoming wind speed, wind direction, and turbulence, as well as turbine operation and associated parameters. In convective conditions, wakes tend to be eroded rapidly by ambient turbulence^4–6^. In stably stratified conditions with weak turbulence, wakes tend to exhibit a large wind speed deficit and persist for long distances downwind^7,8^. For example, aggregated wakes from multiple turbines, i.e., wind plant wakes, can extend more than 50 km downwind of a wind plant, offshore in stable conditions^9^. In addition to the wind speed deficit and the enhancement of turbulence at the heights above the surface corresponding to the wind turbine rotor, wakes can affect local surface conditions. For example, nocturnal surface temperatures can rise because of turbine-induced mixing of the nocturnal inversion^10–12^.

Long-term meteorological measurements in the vicinity of wind plants can also be affected by wind plant wakes. Just as the “urban heat island” effect affects meteorological measurements in and near cities and towns^13,14^, the “wind plant wake” effect can affect measurements of winds, turbulence, and temperature near wind plants^9,11,15^. But the effects of the wakes on local meteorological measurements are generally difficult to discern because the magnitude and orientation of the wind plant wake change with wind speed, wind direction, and atmospheric stability as well as the height of the boundary layer^8,16^; therefore, an accurate assessment of the effects of wind plants on local atmospheric conditions, both aloft and at the surface, is of primary importance for wind plant design^17^, operational management^18^ and for assessing applicability of and uncertainty in the use of meteorological observations near a wind plant. In addition, the importance of such an accurate characterization will continue to increase because wind energy deployment is expected to keep increasing worldwide. In fact, the global installed wind capacity experienced an unprecedented increase by 91% from 2012 to 2017. Future estimates predict an additional growth by a factor of 5 by 2035^19^ and a factor of 10 by 2050^20^, which will lead the wind energy industry reaching the trillion-dollar scale.

The US Department of Energy’s Atmospheric Radiation Measurement (ARM) program’s Southern Great Plains (SGP) site, near Lamont, Oklahoma, is an optimal candidate location for such an assessment. In fact, the site offers a unique array of long-term atmospheric instrumentation. Further, it has been gradually surrounded by wind plants over the last 15 years. Several studies have analyzed the wind flow at the SGP, for example, by looking at the frequent low-level jets^21,22^ or by proposing improved wind speed extrapolation techniques^23,24^. The Lower Atmospheric Boundary Layer Experiment (LABLE) short-term intensive field campaign^25^ has led to better characterization of the boundary layer processes at the site^26,27^. Baidya Roy et al.^10^ simulated the effect of a hypothetical wind plant in the region using short-term coarse-resolution Regional Atmospheric Modeling System (RAMS) mesoscale simulations^28^. However, recent studies^29^ stressed the importance of using mesoscale models at finer horizontal and vertical resolutions for accurately assessing wind plant impacts. In addition, the vast array of long-term instrumentation at SGP offers a unique opportunity to compare simulated wind plant effects with what is seen in real world observations. Therefore, in our analysis, to identify the effects of wakes on longer-term meteorological conditions at the SGP, we first consult a nine-day simulation of wind plant wakes using the state-of-the-art Weather Research and Forecasting (WRF) mesoscale model’s^30^ wind farm parameterization (WFP)^31^ to identify the meteorological characteristics of the wake that might be expected from the wind plants near the ARM facility. Next, we examine observations from ARM instrumentation from 2010 to 2020 to check for such wake signatures.

## Results

### Quantifying wind plant impacts on atmospheric conditions

**Figure 1:**
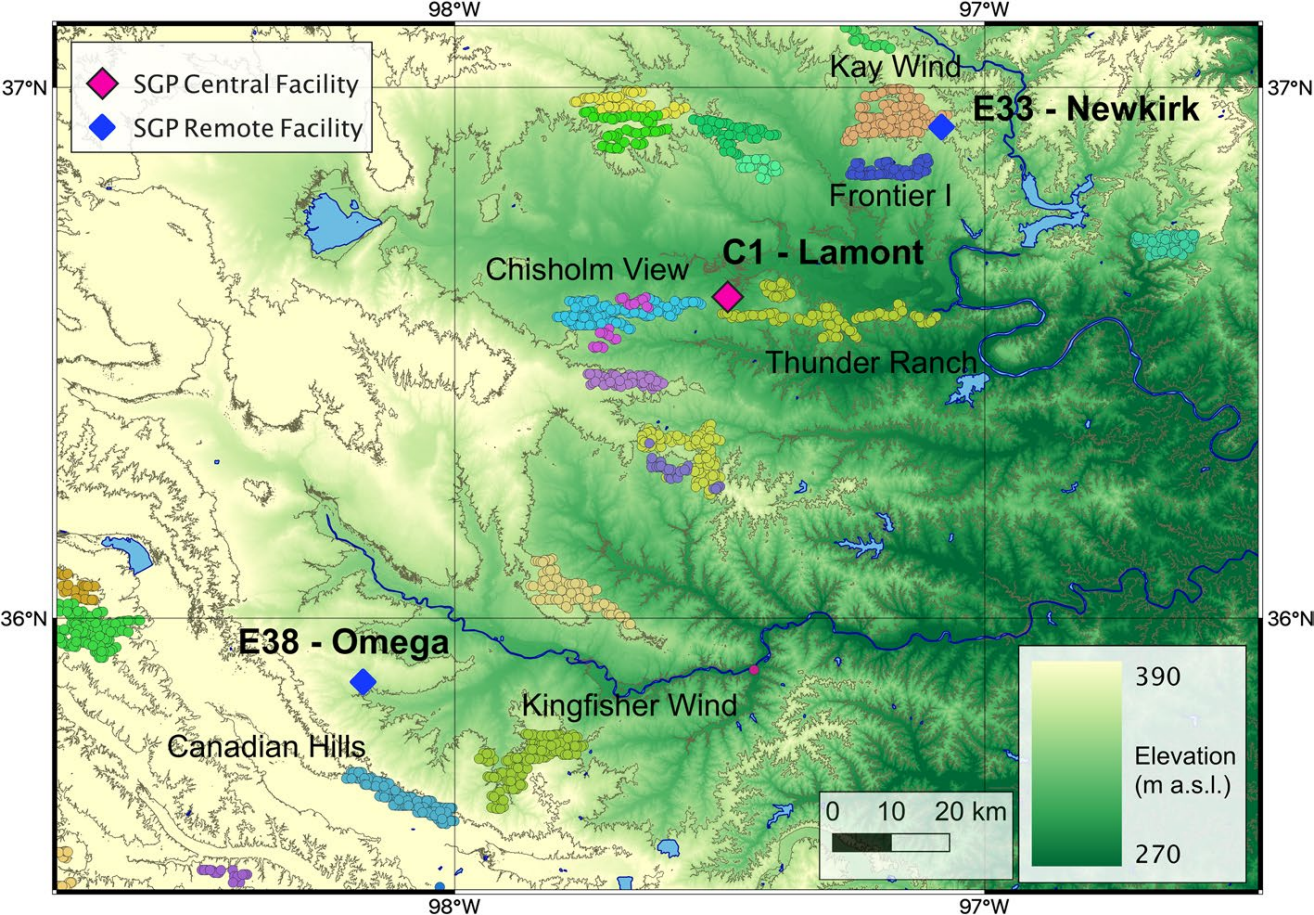
Map of the region and the three measurement sites whose observations are considered in the analysis. The clusters of differently colored dots show wind plants in the region. The named wind plants are those listed in Table 1 and relevant to this analysis. Map created by the authors using the QGIS v3.10.14 open-source software (qgis.org). Digital elevation model data courtesy of the US Geological Survey.

**Figure 2:**
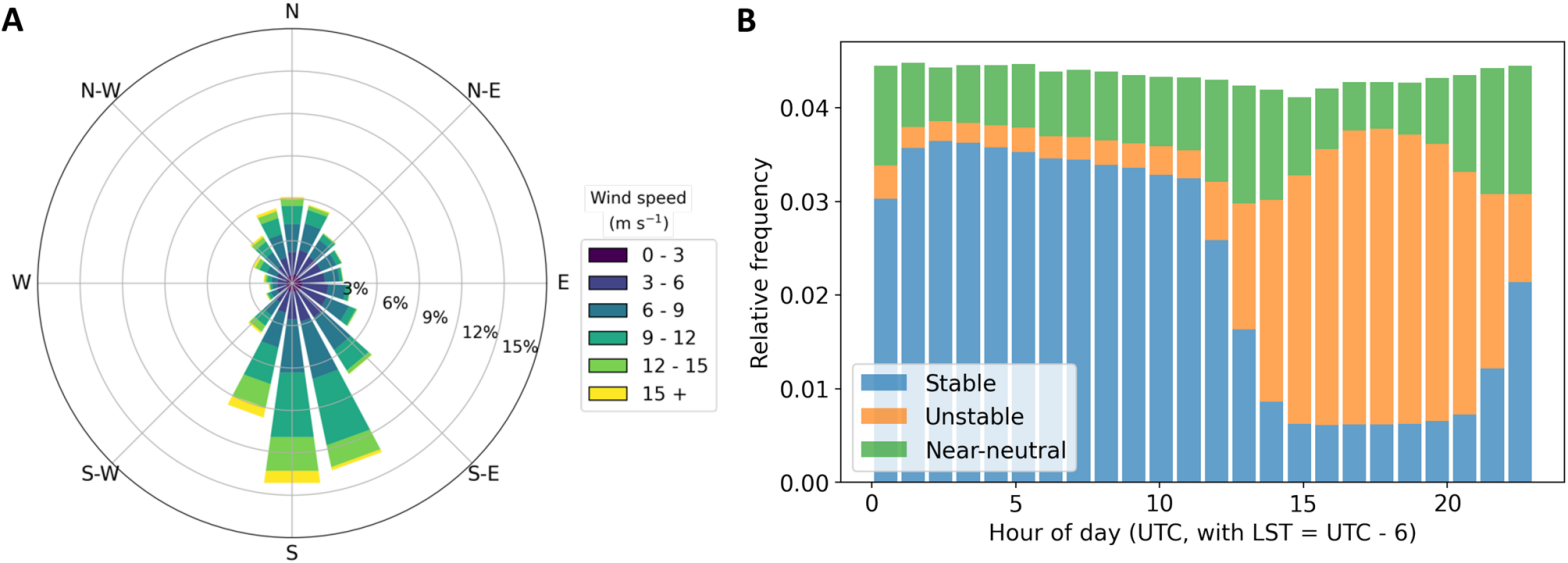
(**A**) 91-m wind rose from the C1 lidar using observations from September 2012 to October 2020. (**B**) Histogram of the atmospheric stability conditions (classified in terms of the surface Obukhov length) as a function of the hour of the day at the C1 site. Data from September 2011 to October 2019.

For the observational component of our analysis, we focus on measurements collected at three sites (see map in Fig. 1): Lamont (C1), Newkirk (E33), and Omega (E38). At the SGP, southerly winds are the most dominant wind regime, followed by winds flowing from the north. On the other hand, easterly and westerly winds are extremely rare, as shown in the wind rose in Fig. 2A, obtained using 8 years of hub-height wind speed observations at the C1 site. Given this wind regime and the relative distances between measurement sites and nearby wind plants (details are included in Table 1), for the observational analysis we focus only on assessing the impacts of the southern portion of the Thunder Ranch wind plant at the C1 site and of Kay Wind at the E33 site. In fact, given the dominant wind regimes, the C1 and E33 measurement sites are often downwind of these two wind plants, and their relatively short distances from the closest turbines allows for significant wake effects to be expected at the sites.

**Table 1:** Main characteristics of the wind plants in the vicinity of the sites considered in the analysis.

Site	Impacting wind plant	Year built	Hub height (m)	Rotor diameter (m)	Distance from closest turbine (km)	Waked wind direction sector
C1—Lamont	Thunder Ranch	2017	90	116	3.5	112$^{\circ }$–196$^{\circ }$
					6.7	67$^{\circ }$–93$^{\circ }$
	Chisholm View	2012	80	82.5	4.6	243$^{\circ }$–270$^{\circ }$
E33—Newkirk	Kay Wind	2015	80	108	1.3	252$^{\circ }$–23$^{\circ }$
	Frontier I	2016	87	126	7.1	195$^{\circ }$–235$^{\circ }$
E38—Omega	Canadian Hills	2012	80	102	18.5	–
	Kingfisher Wind	2015	80	100	25.5	–

To quantify the wind plant impacts using atmospheric observations, we consider 8 years of data collected by both remote sensing and in situ instruments. At C1, we use 30-min average wind speed measurements from a Halo Streamline lidar^32^ from 2012 to 2020. We use three-dimensional sonic anemometers on flux measurement systems deployed at each of the three considered measurement sites from 2011 to 2019 to measure near-surface (4 m AGL) wind speed and turbulence kinetic energy (TKE). Also, we use the observed surface-layer fluxes to quantify atmospheric stability at each site in terms of the Obukhov length, *L*. The distribution of the considered stability classes at C1 as a function of hour of day for the full 8-year period is shown in Fig. 2B.

To more generally explore the expected impact of nearby wind plants we use numerical simulations, comparing 7 days of WRF simulations at the SGP site with and without a WFP^31,33–35^. The parameterization has two impacts. First, it extracts kinetic energy from the mean flow via a drag or momentum sink term in the momentum equations of WRF, based on the hub-height wind speed. Second, it explicitly adds TKE (to that parameterized in the MYNN scheme for subgrid fluxes) at the model levels intersecting the wind turbine rotor disk. In our present analysis, we conduct three sets of simulations, including one with no turbines (without WFP). The two sets of simulations with turbines include either the original TKE source (WFP) or 25% of the original TKE source (WFP25), following indications within the scientific debate on the presence and magnitude of the TKE source in wind plant parameterizations^35–39^. We consider the variability of the results between the two parameterization setups (WFP and WFP25) as a proxy for the uncertainty (especially in TKE changes) resulting from the specifics of the WFP. To this regard, we note how the parameterization proposed by Volker et al.^37^ is not publicly available, and therefore a direct comparison of the results between different WFPs was not possible.

### Wind plant effects on wind speed

**Figure 3:**
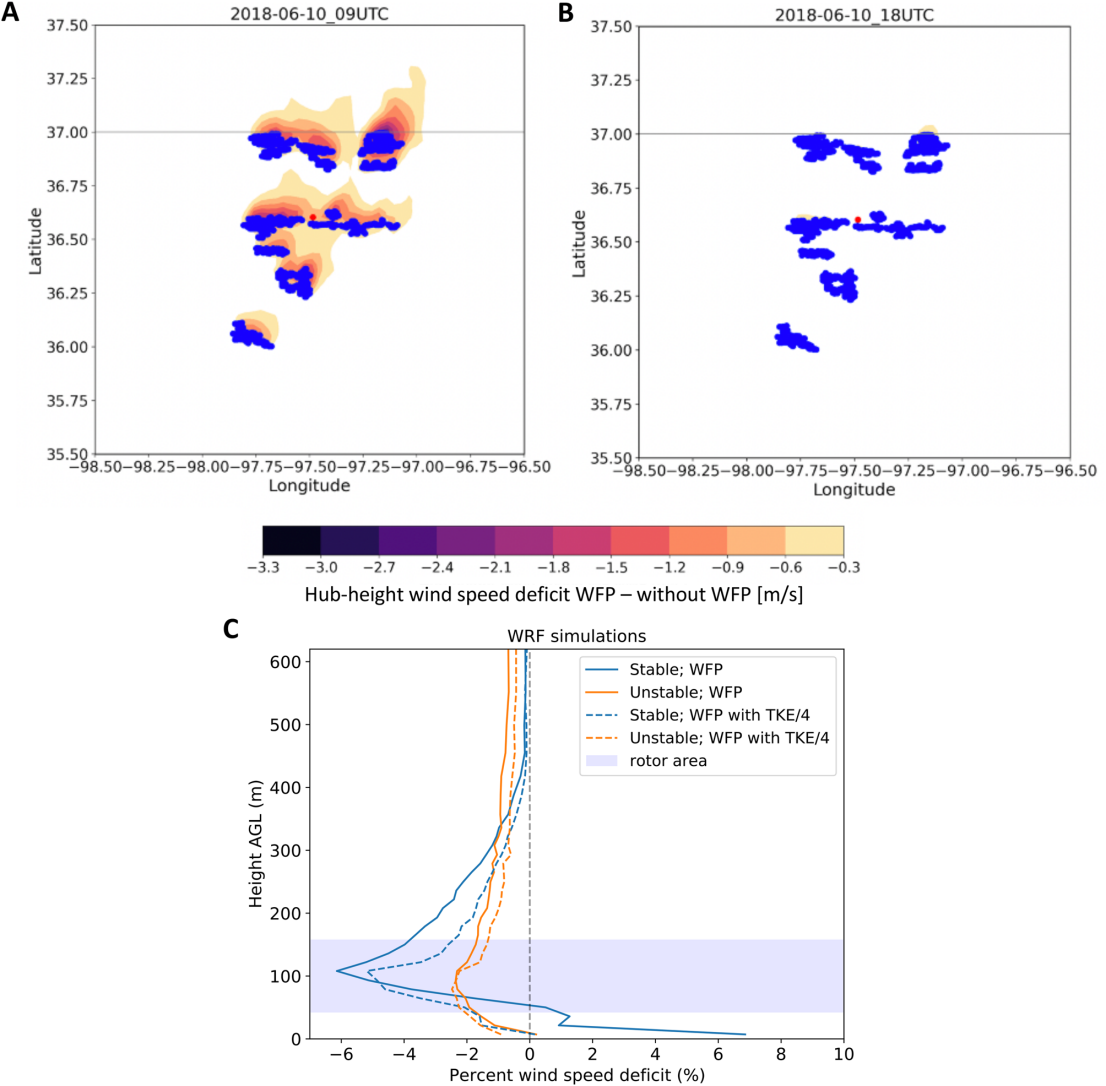
(**A**, **B**) Contours of hub-height wind speed deficit calculated by subtracting the simulation without the WFP from the WFP simulation at two sample times on June 10: (**A**) 0900 UTC (0300 LT); (**B**) 1800 UTC (1200 LT); blue dots represent wind turbines. (**c**) Median profile of WRF-simulated percentage wind speed deficit (calculated as either (WFP − without WFP)/(without WFP) or (WFP25 − without WFP)/(without WFP)) at the C1 location for stable (negative surface heat flux) and unstable (positive surface heat flux) conditions for wind directions between $112^{\circ }$ and $196^{\circ }$.

As shown in other simulation studies^29,40^, the presence of the wind plant induces a wake that varies with wind speed, wind direction, and ambient turbulence. In the “Supplementary Materials”, we include an animation of how the WRF-modeled wind plant wakes vary throughout the considered period. Contours of the wake wind speed deficit at two sample times are shown in Fig. 3A,B. The magnitude of the wake increases as wind speed increases toward the rated wind speed of the turbines; the wake erodes quickly in daytime convective conditions (Fig. 3B) but persists for long distances downwind in stable conditions (Fig. 3A). To further validate this qualitative variability, we compute an average vertical profile of the wind speed deficit at the C1 site in different stability conditions. We calculate the percentage wake wind speed deficit at the C1 site by first subtracting the results from the simulation with no wind plants from the simulations with wind plants (either WFP or WFP25), which is then normalized by the results without the WFP. Also, we select only wind speeds when turbines are expected to be operational (wind speed at $\sim 90$ m above ground level (AGL) between 3 and 25 m ${\text {s}}^{-1}$) and when wind directions are expected to impose wake impacts from Thunder Ranch on the C1 site (simulated wind directions between $112^{\circ }$ and $196^{\circ }$). To check the effects of atmospheric stability, we separate data using the sign of the surface heat flux as simulated by WRF at the location of the C1 lidar; we consider stable conditions for negative surface heat flux and unstable conditions for positive surface heat flux. The median profile of the simulated wake wind speed percentage deficits at the C1 lidar location is shown in Fig. 3C (the median profile of the actual wind speed deficit is included in the “Supplementary Materials” in Fig. S3). The median profile confirms that at most altitudes, the strongest wakes occur in stable conditions for both the WFP and WFP25 cases. Near the surface, accelerations occur in stably stratified conditions but only for the simulations with 100% TKE included. The weaker TKE source (WFP25) shows no accelerations near the surface. Both simulations show a maximum wind speed deficit in the top half of the wind turbine rotor disk but with some detectable deficit persisting up to nearly 400 m above the surface. In these simulations, estimates of stable boundary layer height (not shown) range from 100 to 500 m above the surface. In unstable conditions, both the WFP and WFP25 simulations exhibit a weaker wind speed deficit than in stable conditions, with the maximum wind speed deficit near hub height. Although the unstable wind speed deficit is weaker than that of the stable conditions, it is also detectable at higher altitudes, likely caused by convective mixing throughout the deeper convective boundary layer.

Similar effects emerge from the long-term atmospheric observations. First, we consider measurements aloft using the lidar measurements at C1 to quantify the impact that the southern portion of the Thunder Ranch wind plant has on the long-term lidar data. Although the use of numerical simulations allows for the concurrent availability of data with and without wind plants, this is obviously not the case when looking at real-world observations; therefore, to quantify the wind speed deficit, we calculate at each lidar measurement height, *z*, a normalized median difference in wind speed between the post- and pre-wind plant periods (pre-wind plant data include 2012–2016, post-wind plant data include 2018–2020):

1 $$\begin{aligned} {\text {diff}}_{{\text {WS}},z} = \frac{{\text {median}}({\text {WS}}_{{\text {post}},z})-{\text {median}}({\text {WS}}_{{\text {pre}},z})}{{\text {median}}({\text {WS}}_{{\text {pre}},z})} \end{aligned}$$

**Figure 4:**
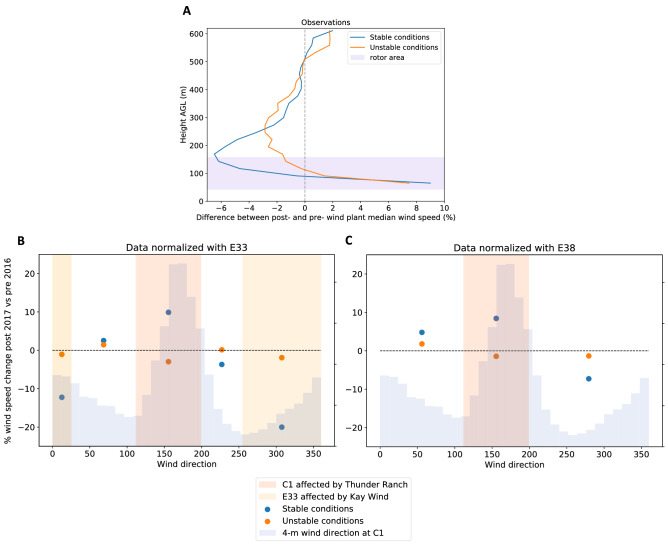
(**A**) Median wind speed percentage deficit between post- and pre-wind plant periods calculated from the lidar observations at C1 for stable and unstable conditions. The blue shaded area shows the vertical limits of the turbine rotor disks (which have been corrected for the $<10$-m terrain elevation difference between the lidar location and the Thunder Ranch wind plant). (**B**, **C**) Median percentage change in 4-m wind speed when comparing post- and pre-wind plant observations as a function of wind direction. Data at C1 are normalized according to Eq. (2) using observations at E33 (**B**) and E38 (**C**). In each panel, the background blue shading indicates the wind direction distribution.

We consider only cases with observed 90-m wind speeds between 3 and 25 m ${\text {s}}^{-1}$ and wind directions between $112^{\circ }$ and $196^{\circ }$, as explained for the WRF case. Figure 4A shows how the median observed wind speed percentage deficit varies with height for stable and unstable conditions (actual wind speed deficit shown in Fig. S4a). The shape of the wake’s vertical profile from the long-term lidar observations generally agrees well with the short-term WRF simulation results (as shown in Fig. 3C), although with some interesting differences in the profile. Both observations and WRF simulations confirm that the wake deficit is stronger in stable conditions, and the magnitude of the observed deficit better agrees with the simulated WFP case than the WFP25 case; however, the peak of the observed wind speed deficit occurs at higher altitudes than the peak in either of the WRF simulations. In fact, although both the WFP and WFP25 simulations showed a maximum wind speed deficit at hub height, the lidar observations display a significant lifting of the location of these peaks. In stable conditions, the altitude of the maximum deficit is near the top of the turbine rotor disk, 75 m above the maximum deficit in the simulations; in convective conditions, the maximum deficit is lofted $\sim 150$ m above the altitude of the maximum in the simulations. This lofting of the wake in convective conditions is consistent with observations^41^ in complex terrain and should therefore be explored further at the SGP site to assess if terrain effects, perhaps unresolved by the model simulations, induce this effect. In addition, the long-term observations reveal a strong acceleration (up to $+10$%) in both stable and unstable conditions. The near-surface acceleration emerges only in stable conditions for the WRF simulations with the WFP and not at all in the WFP25 simulations. Further, the observed accelerations occur at higher altitudes than the WRF results. Accelerations near the surface in wake regions also occur in large-eddy simulations of wind plants^38^. To further explore the near-surface acceleration caused by the wind plant wake, we compare post- and pre-wind plant wind speeds observed from the 4-m sonic anemometers deployed at the three measurement sites considered. The existence of concurrent observations at three locations allows for a further normalization of the wind speed difference to reduce the impact of the wind resource interannual variability; therefore, for the near-surface observations, we modify Eq. (1) and take the difference of the observations at the C1 site with data from either E33 or E38, so the normalized wind speed difference metric becomes (when using E33 for the normalization):

2 $$\begin{aligned} {\text {diff}}_{{\text {WS}},4m} = \frac{{\text {median}}({\text {WS}}_{{\text {post,C1}},4m}-{\text {WS}}_{{\text {post,E33}},4m})-{\text {median}}({\text {WS}}_{{\text {pre,C1}},4m}-{\text {WS}}_{{\text {pre,E33}},4m})}{{\text {median}}({\text {WS}}_{{\text {pre,C1}},4m})} \end{aligned}$$

In this calculation, pre-wind plant data are from 2011 to 2014 (because the Kay Wind wind plant, near E33, was built in 2015), and post-wind plant data include 2018 and 2019 (i.e., after both Kay Wind and Thunder Ranch were built). Figure 4B,C show how ${\text {diff}}_{{\text {WS}},4m}$ varies as a function of wind direction using both E33 (Fig. 4B) and E38 (Fig. 4C) for the normalization of the metric. Corresponding plots for the actual (vs percent) changes in wind speed are shown in the “Supplementary Materials”. In both cases, for the wind direction sector in which the Thunder Ranch wind plant is upwind at C1 (rose shaded area in both Fig. 4B,C), we see how after the wind plant was built, the surface wind speed is nearly 10% larger in stable conditions (blue dots), whereas little change is observed in unstable conditions (orange dots) when strong turbulence quickly erodes wakes. Similarly, when the E33 site is in the wake of the Kay Wind wind plant (yellow shaded area in Fig. 4B), post-wind plant surface wind speed increases by more than 10–20% (which appears as negative values in the figure because of how the normalization was defined in Eq. (2)), whereas no significant changes are observed in unstable conditions. On the other hand, for wind direction sectors not affected by wind plants (white areas in both panels Fig. 4B,C), no significant change in surface wind speed occurs in either stable or unstable conditions in the post-wind plant period.

### Wind plant effects on turbulence kinetic energy

**Figure 5:**
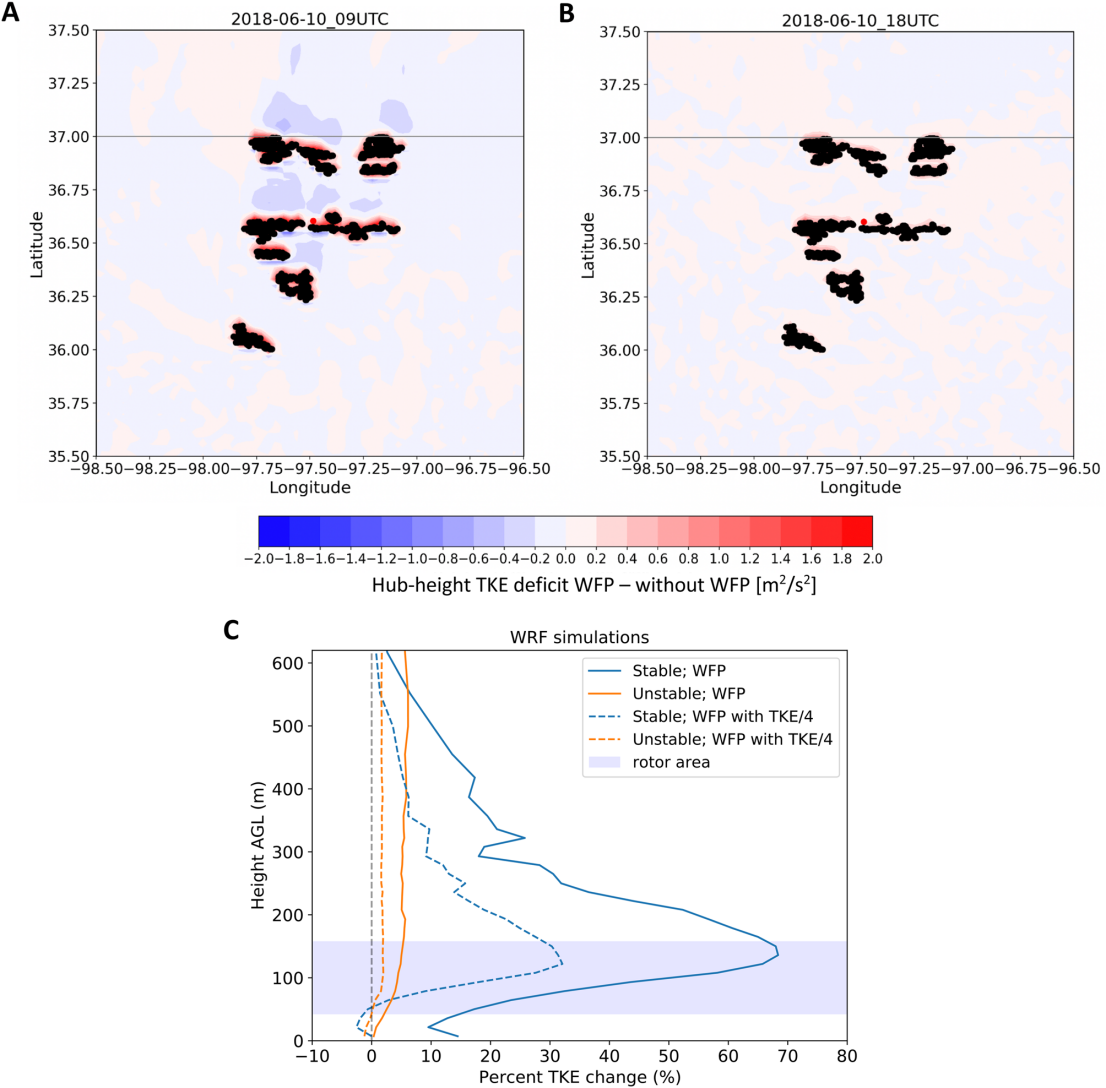
(**A**, **B**) Contours of the difference in hub-height TKE calculated by subtracting the simulation without WFP from the WFP simulation at two sample times on June 10: (**A**) stable conditions, at 0900 UTC (0300 LT); (**B**) unstable conditions, at 1800 UTC (1200 LT); black dots represent wind turbines. (**C**) Median profile of WRF-simulated percentage TKE change (calculated as either (WFP − without WFP)/(without WFP) or (WFP25 − without WFP)/(without WFP)) at the C1 location for stable (negative surface heat flux) and unstable (positive surface heat flux) conditions for wind directions between $112^{\circ }$ and $196^{\circ }$.

TKE is generated by wind turbines as they extract momentum from the wind flow, so it represents another major impact of wind plants on the local atmospheric flow. As shown in the WRF-simulated results in Fig. 5A,B, the immediate vicinity of the wind turbines always exhibits a large positive increase in TKE, but that increase erodes rapidly downwind. In stable conditions (Fig. 5A), the simulation with wakes shows reduced TKE in far downwind regions, likely because of reduced wind shear in the WFP simulation, as discussed in^42^. To generalize this qualitative impact from the WRF simulations, we follow the same approach used to assess the wind speed deficit, and we calculate the percentage TKE change at the C1 site by comparing the simulations without wind plants to those with wind plants (WFP and WFP25). As done for wind speed, we consider only cases where hub-height simulated wind speed is between 3 and 25 m ${\text {s}}^{-1}$ and when the C1 location is directly downwind of the Thunder Ranch wind plant. Figure 5C shows the median profile of the WRF-simulated percentage TKE change at C1 (actual TKE change shown in Fig. S5, along with median TKE profiles in Fig. S6). Maximum TKE enhancements occur at the C1 lidar during stable conditions. In both WRF WFP setups, the peak TKE change emerges in the upper half of the turbine rotor area. On the other hand, TKE is only slightly enhanced during unstable conditions, which are already very turbulent because of surface convective heating. Also, this slight TKE enhancement is more uniform throughout the considered height range, which is consistent with the increased turbulent mixing in convective conditions. At the surface, a significant increase in TKE emerges only in stable conditions for the WFP WRF setup.

**Figure 6:**
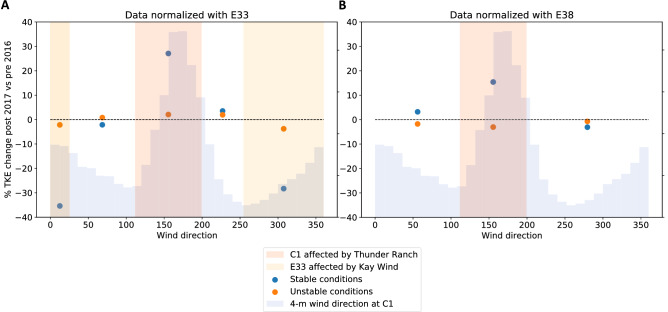
(**A**, **B**) Median percentage change in 4-m TKE when comparing post- and pre-wind plant observations as a function of wind direction. Data at C1 are normalized according to Eq. (2) (but applied to TKE instead of wind speed) using observations at E33 (**A**) and E38 (**B**). In each panel, the background blue shading indicates the wind direction distribution.

No long-term observations of TKE at hub height are available at the SGP site. Still, we can compare the WRF results with the variability in long-term near-surface observations of TKE. Figure 6 shows how 4-m AGL TKE varies between post- and pre-wind plant regimes as a function of wind direction using the same normalized difference metric introduced for wind speed in Eq. (2) (actual TKE change is shown in Fig. S7). Panel A normalizes C1 by the E33 differences; panel B normalizes C1 by E38. The range of wind directions in which wind plant impacts are expected at C1 are highlighted in rose, whereas the range of wind directions in which wind plant impacts are expected at E33 are highlighted in yellow. In both cases, the surface TKE in stable conditions (blue dots) is enhanced by 15–30% when wind plants are located upwind of the measurement site (again, we see negative values in the yellow wind direction sector because of how the normalization was defined in Eq. (2)). On the other hand, in unstable conditions, few differences are detectable between the post- and pre-wind plant conditions when wake erosion is significantly faster. Both results are consistent with those found from the WRF simulations, although the observed increase in surface TKE is greater than what WRF predicts close to the ground. Finally, for wind directions not directly affected by wind plant wakes (white vertical bands in the plots), no changes in observed surface TKE between the post- and pre-wind plant regimes can be detected. We note that we have investigated the variability of upwind terrain elevation at the C1 site for all wind directions (see Fig. S8 in the “Supplement”), and did not find any higher changes in terrain roughness for southerly flow, which could have also caused the increased near-surface turbulence noticed here.

## Discussion

Changing environments can have a significant impact on nearby long-term weather and climate observatories. In cities and towns, the urban heat island effect^13,14^ is well known to affect local atmospheric conditions. Here, we assessed the wind plant wake effect on local atmospheric conditions at the U.S. Department of Energy’s ARM SGP site in northern Oklahoma. Recent increases in wind energy deployment near this and other long-term weather and climate observatories suggest that it is critical to quantify the likely impacts of these changes in effective roughness and mixing on weather and climate observations. Our simulations include a short time period (7 days) of primarily southerly winds. Our observational record considers data from 2011 to 2020. Both simulations and observations show that at the ARM SGP C1 site, approximately 3.5 km downwind of a row of wind turbines, wind speed at wind turbine rotor altitudes decreases by up to 6% in stable conditions, whereas unstable conditions experience a subtle wind speed deficit, not larger than 2%. Near the surface, both the local observations and WRF simulations including a 100% TKE source reveal a significant ($\sim 10$%) acceleration during stable conditions. At the location of the ARM SGP C1 site, TKE is also highly affected by the presence of wind plants. In stable conditions, TKE greatly increases in the WRF simulations, with the maximum change near the top of the turbine rotor disks. Long-term observations confirm this variability, with significant increases ($+30$%) in surface TKE in stable conditions and little change in unstable conditions when boundary layer turbulence values are already large as a result of convection driven by surface heating. Other meteorological parameters can be influenced by wind plant wakes. Both in situ and satellite observations have suggested that nocturnal surface temperatures increase in the vicinity of wind plants as wind turbines mix warmer air from aloft down to the surface. The long-term temperature analysis at this site is complicated by the changing surface cover through the years available for study, by shifts in the timing of transitions in the boundary layer caused by the presence of the wind plants^43^, as well as by the confounding effects of subtle variations in local terrain^44^. Subsequent analysis could explore wind plant wake effects on sensible and latent heat fluxes. Also, a similar analysis could be replicated for different topographic conditions, ranging from offshore to more complex terrain on land.

## Methods

### Area of analysis

The SGP site is a long-term atmospheric observatory managed by the U.S. Department of Energy’s ARM research facility in north-central Oklahoma (Fig. 1). The rural region is characterized by relatively simple topography, and its land use is primarily cattle pasture and wheat fields. The median Terrain Ruggedness Index^45^ calculated over the region around the SGP Central Facility using a 1/3 arc-second Digital Elevation Model is $\sim 0.2$ m. Many wind plants have been built in the area in recent years. They are highlighted by the colored dots in Fig. 1. The C1 site represents the heart of the array of observational equipment at the SGP site, and it has two wind plants in its vicinity: Thunder Ranch to the south and east (yellow-green dots in Fig. 1) and Chisholm View to the west (light blue dots in Fig. 1). The E33 site is approximately 35 km northeast of C1, near the Kay Wind wind plant (salmon dots in the map) and the Frontier I wind plant (blue dots in the map). Finally, E38, approximately 65 km southwest of C1, is generally unaffected by the wind plants in the region, which are at least 18 km from the measurement site. Table 1 includes details of the wind plants in the vicinity of the measurement sites, their mutual minimum distance, and the wind direction sectors for which the measurement sites are downwind of the wind plants.

### Observational equipment

At C1, we use wind speed measurements from a Halo Streamline lidar^32^ from September 2012 to October 2020. Wind speed data from the lidar are retrieved from the line-of-sight velocity recorded during the full 360$^{\circ }$ conical scans, which were performed every $\sim 10$–15 min and took approximately 1 min each to complete. Wind speed is retrieved using the approach described in^46^ by assuming horizontal homogeneity over the scanning volume. Data are then averaged at a 30-min resolution. The instrument operated with a range gate resolution of 30 m, and the lowest height available was 65 m AGL. We discard from the analysis measurements with a signal-to-noise ratio less than $-21$ dB or greater than $+5$ dB (to filter out fog events, relatively common at SGP), together with periods of precipitation, as recorded by a disdrometer at C1. Additional technical specifications of the lidar are shown in Table 2.

**Table 2:** Main technical specifications of the C1 Halo lidar.

Wavelength	$1.5 \; {\upmu }$m
Laser pulse width	150 ns
Pulse rate	15 kHz
Pulses averaged	20,000
Points per range gate	10
Range-gate resolution	30 m
Minimum range gate	15 m
Number of range gates	200

We use in situ instruments to measure near-surface (4-m AGL) wind speed and TKE. We consider data from three-dimensional sonic anemometers deployed at each of the three considered measurement sites. The instruments provide measurements at 10-Hz resolution, which are then averaged at 30-min intervals. At all sites, we use observations from September 2011 to October 2019, the longest period where concurrent measurements from all three locations are available. TKE is provided as a 30-min average, and it is calculated from the variance of the three components of the wind flow as

3 $$\begin{aligned} {\text {TKE}} = \frac{1}{2} (\sigma _u^2 + \sigma _v^2 + \sigma _w^2 ). \end{aligned}$$

Also, we use surface data to quantify the atmospheric stability at each site in terms of the Obukhov length *L*:

4 $$\begin{aligned} {\text {L}}=- \frac{\overline{T_v} \cdot u_*^3}{k \cdot g \cdot \overline{w'T_v '}} \end{aligned}$$

where $k=0.4$ is the von Kármán constant; $g=9.81$ m s$^{-2}$ is the gravity acceleration; $T_v$ is the virtual temperature (K); $u_*=(\overline{u'w'}^2+\overline{v'w'}^2)^{1/4}$ is the friction velocity (m s$^{-1}$); and $\overline{w' T_v'}$ is the kinematic virtual temperature flux (K m s$^{-1}$). A 30-min averaging period is used for the Reynolds decomposition, a common choice for atmospheric boundary layer calculations^47,48^. We classify stable conditions for $0 \, {\text {m}} < L \le 200$ m ($0 < z/L \le 0.02$), unstable conditions for $-200 \, {\text {m}} \le L < 0$ m ($-0.02 \le L < 0$), and near-neutral otherwise. As done for the lidar data, we discard precipitation periods to remove inaccurate measurements.

### Mesoscale model setup

Our simulations use the WRF model^49^ version 4.2.1 and the WFP^31^. While having long-term WRF simulations at the SGP site would be ideal to match the observational analysis, this is not computationally feasible; therefore, here we focus on a 9-day period in 2018, June 10–18, chosen because of the strong and repeated occurrences of nocturnal low-level jets, as seen in the lidar contours of the wind speed (Fig. S1 in the “Supplement”). We exclude from the analysis June 13–14 because of strong precipitation events in the region, which could affect the accuracy of the WRF predictions^50^. Two one-way nested domains (9-km horizontal resolution with $250 \times 250$ points and 3-km horizontal resolution with $301 \times 301$ points) are simulated, with both domains centered at 36.605$^{\circ }$ N, 97.485$^{\circ }$ W, the location of the C1 site. The 58 vertical levels include eight levels in the lowest 100 m, following best-practice recommendations^29^. Initial and boundary conditions come from the ERA5 reanalysis product^51^. The Rapid Radiative Transfer Model (RRTM) shortwave and longwave radiation schemes^52^ represent radiative processes, whereas a cumulus parameterization is active only on the 9-km domain. The planetary boundary layer scheme is the MYNN scheme^53^, currently the only scheme that functions with the WFP. Each of the 9 days is run separately with 24 h of spin-up time to ensure accurate representation of surface soil moisture^54^.

To represent the effects of wind turbines, we use the WRF WFP. The original version^31^ has experienced several updates and changes^33–35^. The presence and magnitude of the TKE source in the wind plant parameterizations is an area of scientific debate. Some wind plant parameterizations do not explicitly add TKE^36,37^ and allow it to be developed from wind shear caused by the momentum sink. Comparisons to large-eddy simulations^38^ and observations^39^, however, suggest that the TKE source term is critical to include, although some investigators^35^ suggest that the TKE source term should be reduced. Further, the integration of the WFP with boundary layer parameterizations other than the MYNN scheme might support more shear-developed turbulence^55^. In our present analysis, we conduct three sets of simulations, including one with no turbines. The two sets of simulations with turbines include either the original TKE source (WFP) or 25% of the original TKE source (WFP25). WRF namelists and wind turbine supporting files are available at 10.5281/zenodo.4641408.

We include 949 turbines in the simulation; their locations are taken from the US Geological Survey database^56^, and each is represented as a 2-MW turbine with 80-m hub height and 80-m rotor diameter. The actual turbines in this domain all have slightly different capacities (ranging from 1.5 to 2.3 MW), hub heights (ranging from 80 to 90 m), and power curves. Within the WRF simulations, we represent them all with the same power curve with a 3-m ${\text {s}}^{-1}$ cut-in speed, 12-m ${\text {s}}^{-1}$ rated speed, and 25-m ${\text {s}}^{-1}$ cut-out speed (Fig. S2 in the “Supplement”). Other researchers^12^ have investigated the sensitivity of the WFP to exact turbine power curves by varying the wind turbine thrust and power coefficients by 10%. They found that the uncertainty of the resulting wind speed deficit induced by these coefficients was smaller than the uncertainty induced by a suite of other physics and numeric permutations, including a change in the number of vertical levels; thus, we do not use unique turbine characteristics for the turbines in this region. The turbines included in the simulation setup are shown in Fig. 1, except for the two wind plants in the vicinity of site E38, which were omitted from the WRF runs because they do not impact any measurement site. The exact turbine locations are available at 10.5281/zenodo.4641408. When multiple turbines are located within a single grid cell, as often occurs, the drag and TKE source are multiplied by the number of turbines within the cell and integrated over the cell. The amount of drag and TKE is a function of hub-height wind speed rather than rotor-equivalent wind speed, although an option for considering rotor-equivalent wind speed has been developed^34^.

## Supplementary Information

Supplementary Video S1.

Supplementary Video S2.

Supplementary Information.

## Data Availability

WRF namelists and supporting files required to run the WRF simulations in our analysis are available at 10.5281/zenodo.4641408. All the observations used in the analysis are publicly available at the following DOIs: 10.5439/1025186; 10.5439/1025039; 10.5439/1025036; 10.5439/1342140.
