# Supplementary material for: Wind plants can impact long-term local atmospheric conditions
Source: Sci Rep. 2021 Nov 25;11:22939. doi: 10.1038/s41598-021-02089-2 (PMC8617173; doi:10.1038/s41598-021-02089-2)
Supplement: Supplementary file 3 — Supplementary Information. [file 41598_2021_2089_MOESM3_ESM.docx]

**Supplementary materials**

Figs. S1 to S7

Animations S1 to S2


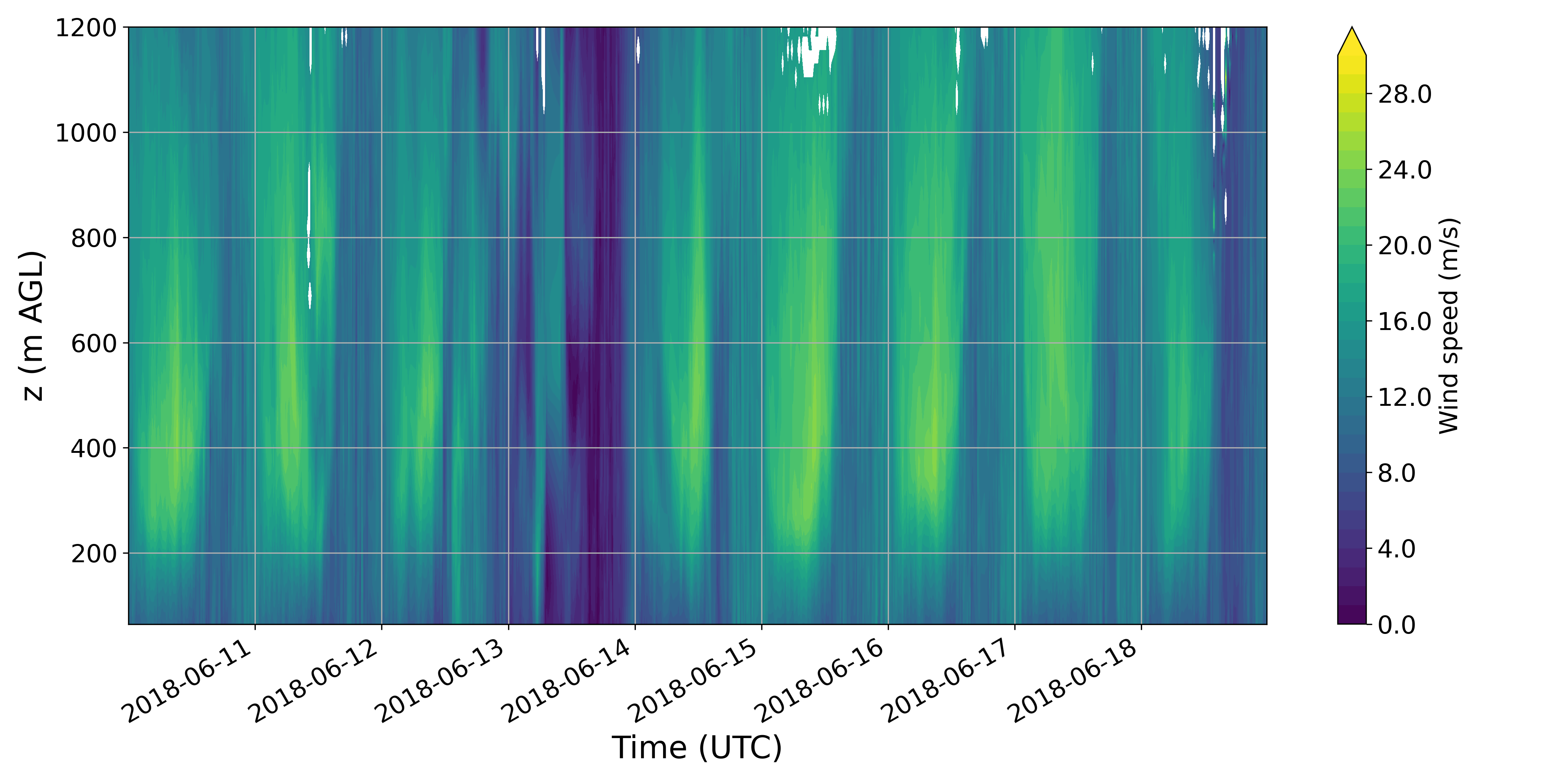


Figure S1: Time-height cross-sections of wind speed from the C1 lidar for 10-20 June 2018, which is the period selected to run the WRF simulations.

Figure S2: Power curve and thrust coefficient (c_T_) curve of the generic 2-MW turbine used in the WRF simulations.

Figure S3: median profile of WRF-simulated wind speed change at the C1 location for stable (negative surface heat flux) and unstable (positive surface heat flux) conditions for wind directions between 112 and 196 deg.

Figure S4: (a) Median wind speed deficit between post- and pre-wind plant periods calculated from the lidar observations at C1 for stable and unstable conditions. The blue shaded area shows the vertical limits of the turbine rotor disks (which have been corrected for the <10-m terrain elevation difference between the lidar location and the Thunder Ranch wind plant). (b--c) Median change in 4-m wind speed when comparing post- and pre-wind plant observations as a function of wind direction. Data at C1 are normalized according to equation 2 in the main paper using observations at E33 (b) and E38 (c). In each panel, the background blue shading indicates the wind direction distribution.

Figure S5: median profile of WRF-simulated TKE change at the C1 location for stable (negative surface heat flux) and unstable (positive surface heat flux) conditions for wind directions between 112 and 196 deg.

Figure S6: median profile of WRF-simulated TKE at the C1 location for stable (negative surface heat flux) and unstable (positive surface heat flux) conditions for wind directions between 112 and 196 deg, for the various WRF setups considered in the analysis.

Figure S7: (a--b) Median change in 4-m TKE when comparing post- and pre-wind plant observations as a function of wind direction. Data at C1 are normalized according to equation 2 in the main paper (but applied to TKE instead of wind speed) using observations at E33 (a) and E38 (b). In each panel, the background blue shading indicates the wind direction distribution.

Figure S8: Standard deviation of upwind terrain elevation for various 5-km radius sectors centered at the SGP C1 location.


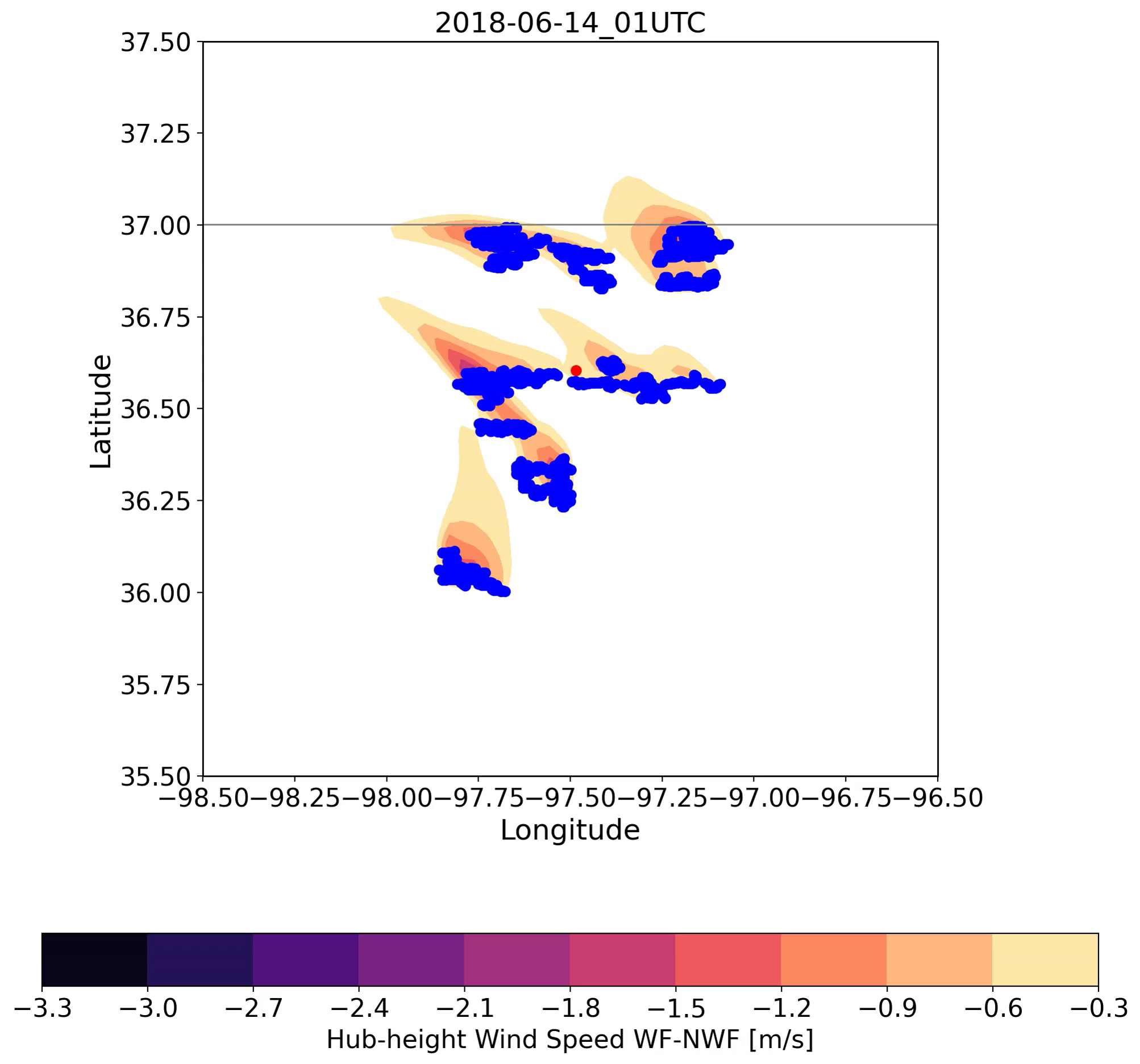


Animation S1: animation of the temporal variability of the hub-height wind speed deficit from the WRF simulations.


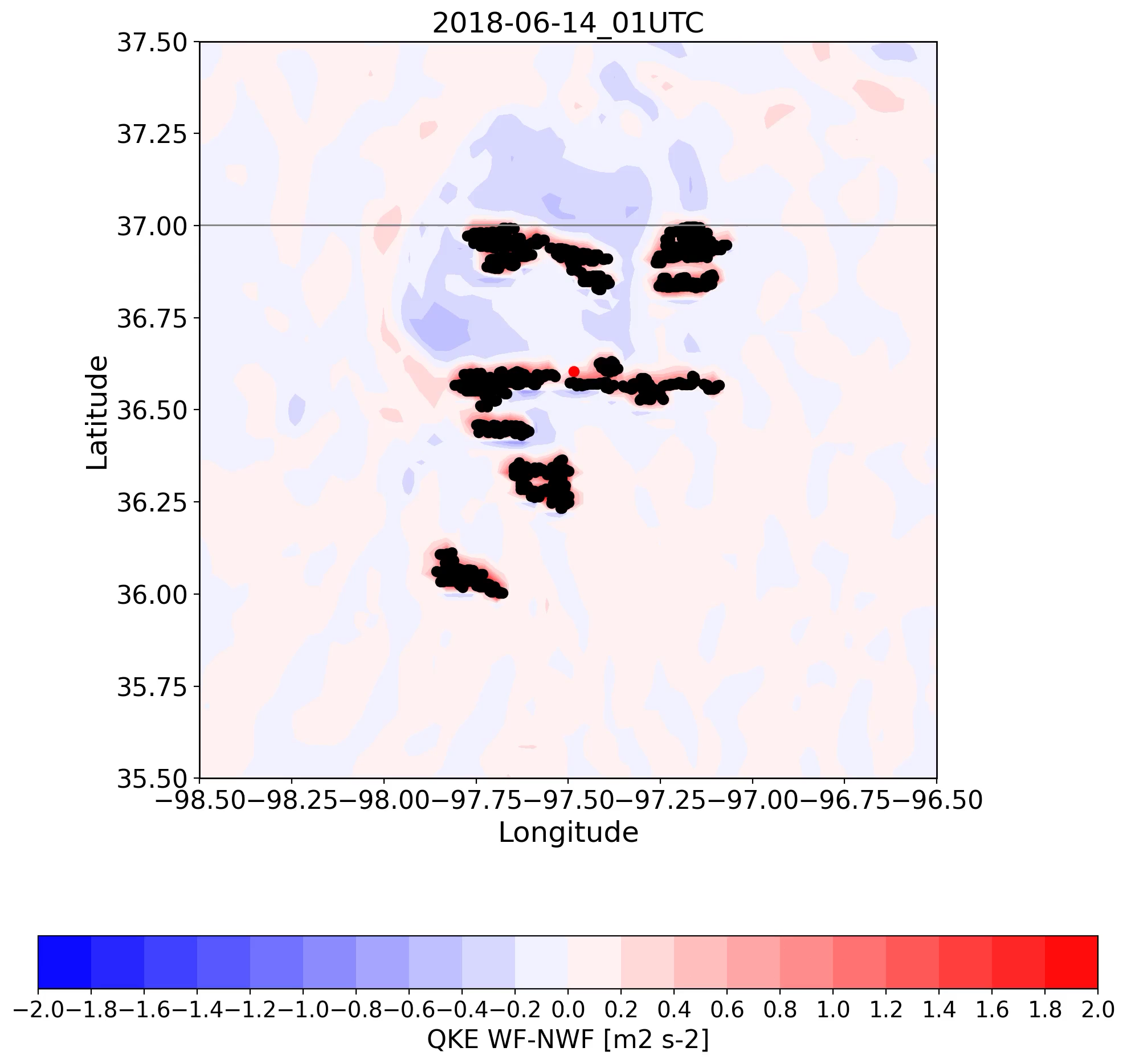


Animation S2: animation of the temporal variability of the hub-height QKE (=2*TKE) deficit from the WRF simulations.
